# Implementing personalised care planning for older people with frailty: a process evaluation of the PROSPER feasibility trial

**DOI:** 10.1186/s12877-022-03426-4

**Published:** 2022-09-16

**Authors:** Nicky Kime, Alan Wright, Anne Heaven, Rebecca Hawkins, Jane Smith, Bonnie Cundill, Robbie Foy, Rebecca Lawton, Amanda Farrin, Claire Hulme, Andrew Clegg

**Affiliations:** 1 Academic Unit for Ageing and Stroke Research, University of Leeds, Bradford Institute for Health Research, Bradford Teaching Hospital NHS Foundation Trust, Bradford, BD9 6RJ West Yorkshire UK; 2 Clinical Trials Research Unit, Leeds Institute of Clinical Trials Research, University of Leeds, West Yorkshire, Leeds, LS2 9JT UK; 3 School of Medicine, Leeds Institute of Health Sciences, University of Leeds, West Yorkshire, Leeds, LS2 9NL UK; 4 School of Psychology, University of Leeds, West Yorkshire, Leeds, LS2 9JT UK; 5 Institute of Health Research, University of Exeter Medical School, St Lukes Campus, Exeter, EX1 2LU UK

**Keywords:** Personalised care planning, Older people, Frailty, Feasibility, Evaluation

## Abstract

**Background:**

Personalised Care Planning (PCP) is a collaborative approach used in the management of chronic conditions. Core components of PCP are shared decision making to achieve joint goal setting and action planning by the clinician and patient. We undertook a process evaluation within the PROSPER feasibility trial to understand how best to implement PCP for older people with frailty in the community.

**Methods:**

The trial was set in two localities in England. We observed training sessions and intervention delivery at three time points during the 12-week intervention period. We interviewed delivery teams before, during and after the intervention period, as well as primary care staff. We interviewed older people who had received, declined or withdrawn from PCP. We explored training of staff delivering PCP, structures, mechanisms and resources needed for delivery, and influences on uptake. We undertook a framework approach to data analysis.

**Findings:**

We observed thirteen training sessions and interviewed seven delivery staff, five primary care staff, and twenty older people, including seven who had declined or withdrawn from the intervention. Delivery teams successfully acquired skills and knowledge, but felt underprepared for working with people with lower levels of frailty. Timing of training was critical and ‘top-ups’ were needed. Engagement with primary care staff was tenuous. Older people with lower frailty were unclear of the intervention purpose and benefits, goal setting and action planning.

**Conclusions:**

PCP has the potential to address the individualised needs of older people with frailty. However, training requires careful tailoring and is ideally on-going. Considerable efforts are required to integrate statutory and voluntary stakeholders, understanding the expectations and contributions of each agency from the outset. In addition, older people with frailty need time and support to adjust to new ways of thinking about their own health now and in the future so they can participate in shared decision making. These key factors will be essential when developing models of care for delivering PCP to support older people with frailty to sustain their independence and quality of life.

**Trial registration:**

ISRCTN 12,363,970 – 08/11/2018.

**Supplementary Information:**

The online version contains supplementary material available at 10.1186/s12877-022-03426-4.

## Background

Personalised Care Planning (PCP) is a “series of discussions between a patient and a health professional to clarify goals, options and preferences and develop an agreed plan of action”. Recognised as giving people greater control over their own health, it is a collaborative process focused on what is important to the individual in the context of their daily lives and has been shown to improve physical and mental health [1]. The core principles of PCP, person centeredness and shared decision-making, have been widely used in the care of people with long-term conditions (LTCs), including well-established models such as the Chronic Care Model [2–4]. Aligned with this, the English National Health Service (NHS), has emphasised PCP in its Long-Term Plan [5], supporting implementation through national funding arrangements [6].

Historically, the focus of PCP has been on single disease states, for example, diabetes [7] or kidney disease [8]. However, the core principles of PCP are also relevant to other important groups including older people living with frailty, a condition characterised by loss of reserves and vulnerability to a range of adverse outcomes, including falls, loss of independence, and care home admission. Older people with frailty generally have multiple long-term conditions (MLTC), representing a subgroup of those living with MLTC at especially high risk of adverse outcomes [9]. Frailty is common, affecting around 12% of people aged 65 and over, rising to around one third of people aged 80 and over [10]. PCP provides an opportunity to a move away from a reactive, disease focused response in frailty towards a more proactive and person-centred approach that widens the traditional health focus of care to a more socially-orientated approach [11, 12]. Moreover, it has been demonstrated that significant social care savings could be achieved if older people did not transition into worsening frailty states [13].

However, PCP has rarely been applied or researched in the context of frailty, and may require initial adaptation to account for the needs of this highly complex group with a range of individual circumstances and predicaments. This is supported by an evaluation of PCP implementation in the UK, which reported universally poor implementation and limited support for people with frailty [14]. The absence of evidence on the effectiveness of PCP for older people with frailty and how best to embed it within existing services is a major evidence gap for healthcare systems internationally.

The Personalised Care Planning for Older People (PROSPER) research programme has been commissioned by the UK National Institute for Health Research to address this important evidence gap. The PROSPER intervention has been co-designed to implement PCP for older people with frailty within primary care, aiming to improve quality of life for people with frailty and reduce use of health and social care services. This paper reports findings from the process evaluation of the PROSPER feasibility trial [15].

## Aims

The process evaluation aimed to examine the implementation of the key components of the PROSPER intervention in practice. The objectives were to:

1. Examine PROSPER staff training and observe skill acquisition and maintenance;
2. Examine how service structures, practices and resources shape PROSPER delivery (contextual factors that shape intervention delivery);
3. Consider what influences PROSPER intervention uptake among older people.

## Methods

### Design

#### Feasibility trial

The PROSPER feasibility trial was a multi-centre, two-arm, cluster Randomised Controlled Trial (RCT) which recruited participants from two large metropolitan districts of West Yorkshire, England, from March 2019 to January 2020. A cluster was defined as a general practice or group of practices where the practices shared significant staff and/or services [15]. Eleven clusters across two localities were randomly assigned to either the intervention or control (usual care) group.

### Implementation

#### Training

Training in PROSPER was conducted over a four week period and comprised key activities in relation to delivery. Activities included background to the PROSPER service, PCP principles, guided conversations, motivational interviewing, behaviour change techniques (BCTs), data management processes, recording and monitoring, research principles, etc. Each training session took place on a different date and varied in length from one hour to a whole day. Sessions were delivered by a combination of professional trainers, for example, motivational interviewing and behaviour change techniques, and staff working on PROSPER, for example, PCP principles and recording and monitoring. Training was conducted either face-to-face or online.

### The intervention

**Fig. 1:**
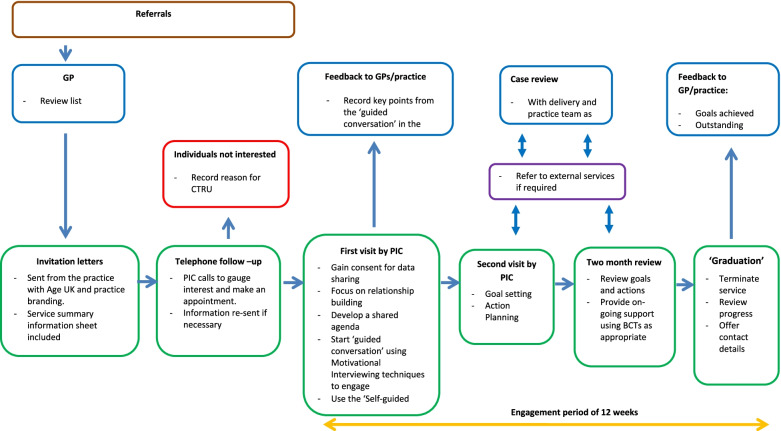
The PROSPER pathway

The PROSPER PCP process aimed to build confidence and instil in older people the knowledge and skills to set personal goals and develop personalised action plans. Two delivery teams comprised Personal Independence Co-ordinators (PICs), support workers and team leaders. The delivery teams were employed by Age UK, a charitable organisation independent of the NHS, but linked to primary care staff in general practices through honorary contracts. The PICs, support workers and team leaders came from a variety of professional backgrounds, including the NHS and voluntary/charitable sector. All had social and/or clinical experience. The PICs employed guided conversations with the older people which supported shared decision making to identify goals, agree action plans and build self-management capabilities. Support workers facilitated practical activities and team leaders provided supervision and support. A detailed description of the PROSPER intervention is provided in Table 1. A flowchart showing the PROSPER pathway is in Fig. 1.

**Table 1:** The PROSPER template for intervention description and replication—TIDieR

Procedures
1. The key components of the intervention are the ‘guided conversation’ and ‘graduation’ as delivered by the Personal Independence Co-ordinator (PIC)
2. Following consent and baseline assessment, intervention practices send letters of invitation to registered participants, branded with both the practice and Age UK logos, to offer the PCP intervention
3. Unless the participant has contacted the surgery to ‘opt out’, the PIC follows-up with a phone call 5–10 days after the letter has been sent out. The purpose of the phone call is to discuss the personalised care planning service in more detail and arrange a convenient time for the first visit
4. Before the first visit the PIC records a minimum amount of personal information from the patient’s Electronic Health Record
5. The first meeting should take place approximately one week after the initial phone call, if possible. The main focus of this visit is on information sharing and relationship building. This visit will;
○ Make it clear that the service is time limited in order to manage expectations
○ Let the older people know that they may receive on-going support from the Support Worker alongside the PIC
○ Note any key contacts
○ Outline what support is and is not on offer and, importantly, when it is on offer i.e. Monday to Friday during office hours
○ Leave the summary information booklet in for the older people to read
○ Complete and leave the quick reference fridge magnet. An expected end date should be recorded on the magnet to remind the older people that contact time is limited
6. A second visit is then scheduled within one week. The focus of the second visit is to co-produce an action plan with older people, through a ‘guided conversation’ which will incorporate ascertaining information about: a usual day; support networks; social networks; mobility and transport; health and fitness; safety and security; finances & paperwork and use of statutory support providers
7. An action plan based on the older people goals is co-produced with the older people. The action plan will record: the goal; importance of the goal; confidence in achievement of the goal; motivation to achieve the goal; enablers to achieve the goal and the target date for achievement of the goal
8. The wider PCP team – PIC and Support Worker—work with the older people to achieve the goals set out in the action plan
9. Information about the older people is fed-back to the primary care Multi-disciplinary Team (MDT) and significant outputs recorded in their electronic health care records (systmone, EMIS, VISION etc.)
10. A review of the Action Plan takes place at two months. Older people are reminded of the expected end date of the intervention
11. A final review takes place at 12 weeks. The current status of goal achievement is recorded; delays in goal achievement due to delays with third parties noted
12. Older people ‘graduate’ from the service after a period of approximately 12 weeks. The ‘graduation’ session provides an opportunity for the older people and PIC workers to discuss future options including a step down in support and routes to re-engagement with services if necessary in the future
**Provider**
1. Screening for eligible older people is undertaken by administrative staff within general practices
2. Potential older people are reviewed by GPs with in-depth knowledge of the practice list. All practice staff receive proportionate GCP training to ensure adherence to the trial protocol
3. Guided conversations and graduations are undertaken by Personal Independence Co-ordinators employed by local Age UK offices
4. Support to the older people during the intervention period is provided by the PIC and Support Worker depending on the specific type of support required. Support may also be provided by friends and family or other statutory or third sector organisations
**Delivery mechanism**
1. The intervention is delivered by a trained team of Personal Independence Co-ordinators
2. The intervention is aimed at an individual but if appropriate an individual’s carer/spouse may be involved in the development of the action plan
3. Specific behavioural change techniques are employed to facilitate the older people in achieving their goals
4. Support is offered face to face or via email and/or telephone, as appropriate
5. Practices will allow PIC workers access to electronic health care records in order to record pertinent information for the MDT members
6. Practices will hold MDTs at least every 4 weeks in order for the PIC to feedback on action plan progress and raise any specific issues that need to be addressed by other MDT members
**Delivery location**
1. The intervention is delivered in the participants own home, in communities and via the telephone and/or email, where appropriate
**Duration and Intensity**
1. The intervention is delivered over a period of approximately 12 weeks depending on the specific needs of the older people
2. Face to face contact is made at the start (approximately 90 min) and the end of the intervention (approximately 45 min) with telephone follow up and hands on support in achieving goals given if necessary. A two month review of action plan progress is also conducted face to face
**Tailoring**
1. PCP is designed to be person centred and tailored to individual older people needs and circumstances
2. The Action Plan remains a dynamic document and goals may be added/amended throughout the period of engagement

### Process evaluation

Our process evaluation drew upon the Medical Research Council guidance on the evaluation of complex interventions [16], specifically by examining 1) the implementation of key elements of the intervention – for example, the training and delivery of PCP sessions; 2) how contextual features of the primary care and voluntary/charitable sector shaped how the intervention was implemented; 3) experience of older people, delivery teams and primary care staff and their engagement with the intervention. To examine these factors, we adopted a qualitative design [17]. 

### Data collection

Four intervention clusters across the two localities were purposively sampled to ensure variation in relation to configuration of the delivery team, experience of PCP, location (rural/urban) and the demographics of the local population including ethnicity and deprivation. The characteristics of the sampled sites are shown in Table 2.

**Table 2:**
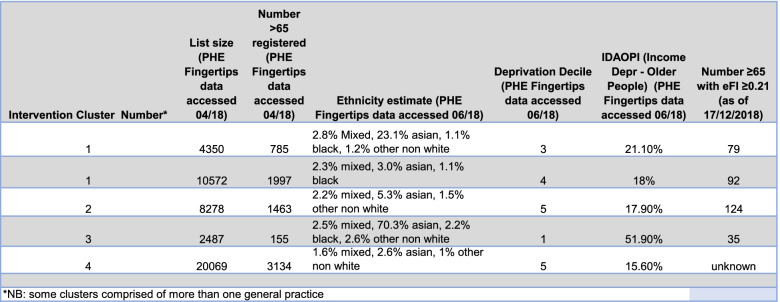
The characteristics of intervention clusters

We undertook observations, semi-structured interviews and reviewed the available trial monitoring data between April and December 2019. See Table 3. Interviews were conducted until data saturation was achieved and no repeat interviews were carried out.

**Table 3:** Process evaluation data collection

Method	Sample number (targets in brackets)	Time-points	Target of data collection
Training observations	13 (13) training sessions	Training delivery	Implementation
Post-training interviews	3 (3) PICs 2 (2) Support Workers 1 (1) Team Leader	Early intervention delivery	Implementation
Guided Conversation observations	17 (20) visits	Older people’s initial contact with service	Intervention delivery and reception by older people
Two-month review observations	2 (16) visits	Older people’s contact with service	Intervention delivery and reception by older people
Two-month review interviews with Delivery Teams	2 (8) PICs	Older people’s contact with service	Intervention delivery and reception by older people
Graduation observations	7 (4) visits	Completion of older people’s contact with service	Intervention delivery and reception by older people
Graduation interviews with Delivery Teams	6 PICs	Completion of older people’s contact with service	Intervention delivery and reception by older people
Older people / carer interviews	20 older people (including 2 carers) (20)	Completion of older people’s contact with service	Reception by older people
End of intervention interviews	3 (3) PICs 2 (2) Support Workers 2 (2) Team Leaders	End of intervention delivery	Implementation
Primary Care Team interviews	3 GPs 2 Practice Managers (12)	End of intervention delivery	Context

#### Observations

The training sessions were observed by members of the process evaluation team, one Senior Research Fellow and two Research Fellows working on PROSPER. They aimed to observe all training sessions (*n* = 13) to gauge acquisition levels through the comprehension of, and engagement with, the content and delivery methods of the training. In addition they intended to observe four to five initial meetings/guided conversations (*n* = 20 max), four two-month reviews (*n* = 16), one ‘graduation’ (*n* = 4) and two Multi-Disciplinary Team (MDT) meetings per cluster across the two localities (*n* = 8). Spradley’s framework [18] guided the observations, which is an approach that prioritises nine topics or foci for those conducting observations. This 9-point guide assists the researcher in areas to observe during interaction. Please see the PROSPER observation guide in the supplementary material.

#### Semi-structured interviews

The process evaluation team intended to interview all members of the delivery teams (*n* = 7) at three time points: after the PROSPER training; following a two-month review or ‘graduation’ with an older person and at the end of the PROSPER intervention period. The purpose of the interviews was to elicit opinions on the following domains: training, referrals, case-loads, information sharing and integrated working with the primary care staff and links to the wider community networks. Interviews were conducted by members of the process evaluation researchers face to face and at either their place of work or that of the delivery teams. Please see topic guides 1–5 used to interview the Age UK delivery teams.

The plan was also to conduct five semi-structured interviews per cluster (*n* = 20) with a purposive sample of older people and their carers face to face in their own homes. Topic guides explored older people’s understanding of intervention aims, their relationship with the PROSPER delivery team, identification of goals, implementation of an action plan and any change in behaviour as a result of PROSPER involvement. Please see topic guides 6 and 7 used to interview the older people.

Two interviews per cluster (*n* = 8) were also planned for primary care staff. These interviews were to elicit information on usual care for individuals with frailty. They also explored the process of referrals, information sharing and integrated working with the PROSPER delivery team, along with the wider organisational implications of embedding and sustaining the PROSPER intervention. Interviews took place face to face at the general practice premises towards the end of the process evaluation to enable participants to reflect on their practice. Please see topic guide 8 used to interview the primary care staff.

Interviews lasted approximately thirty to sixty minutes, were audio-recorded with the participants’ permission and transcribed verbatim.

#### Monitoring data

Trial monitoring data was collected by the PROSPER delivery teams in each of the four intervention clusters using standardised monitoring forms. This data included baseline characteristics of the older people, delivery teams and general practices and data pertaining to training and intervention delivery. Data around participants’ characteristics informed the sample and the process evaluation findings in relation to goal setting.

The descriptive data gathered as part of this monitoring was used to elucidate findings from interviews and observations. Quantitative data describing the dose and reach of the intervention, along with fidelity to specific elements of the protocol, for example, the number of two-month review visits, will be published elsewhere.

### Recruitment and written consent

#### PROSPER delivery teams and primary care staff

Delivery staff from Age UK and primary care staff from the general practices were sampled according to professional role, (for example, PIC, support worker, team leader, General Practitioner (GP), practice manager, etc.) and locality and were approached directly by the process evaluation researchers to participate in the process evaluation. They were provided with an information sheet and a consent form which they were asked to complete and sign to indicate informed consent before they could be recruited to the process evaluation.

#### Older people

All older people who were offered the PROSPER intervention by the delivery team were asked to consent to being contacted about the process evaluation by an independent researcher not involved in baseline or follow-up data collection for the feasibility trial. Consent for the process evaluation was then obtained by the process evaluation researchers and was separate to the consent obtained for the feasibility trial [15]. In addition, carers and significant others were asked for consent to contact. Older people were purposively sampled to ensure maximum variation in relation to characteristics that may shape how the intervention was delivered and engagement with the intervention: frailty score, the electronic Frailty Index (eFI) [19]; gender; age; ethnicity and living circumstances. Confidentiality, anonymity and ethical approval for the process evaluation were assured. All participants were informed that they could obtain a copy of the evaluation report on request.

None of the process evaluation researchers had any knowledge of the participants prior to the evaluation. As part of the data collection process, the researchers sought to establish a rapport with the participants, which included explaining their interest and roles in PROSPER.

### Analysis

A framework approach to data analysis [20] was used to explore implementation of the PROSPER intervention. Data from the interviews and observations with older people, delivery staff from Age UK and primary care staff were each analysed separately and then combined, along with the process evaluation researchers’ reflective diaries and field notes (made during the observations and following the interviews), to inform understanding of the key components that shaped implementation of the PROSPER intervention in practice.

Analysis was concurrent with the data collection phase to ensure that initial themes could be identified and specific areas warranting additional investigation subsequently explored. A sub-set of interview transcripts and observation notes were analysed independently by two of the process evaluation researchers to inform the first iteration of the framework. Interview data was collated and coded according to the original objectives. Further inductive themes were generated and added to the initial framework. The framework was tested on a further sub-set of the data and refined through discussion and consensus before being applied to the remaining data. Analytic summaries of the observation data were combined with the thematic data from the interviews. Patterns within and across delivery teams, primary care staff and older people were identified to understand similarities and differences in relation to implementation processes and individuals’ experiences of the intervention. Following this, themes were summarised and discussed amongst the process evaluation researchers and then further refined. Unpopulated frameworks are available from the corresponding author on request. Data from all participant groups collected through observations and interviews were used to report the findings. Longitudinal data and multiple data collection, through observations and interviews, provided an opportunity to examine and check the data.

## Findings

Delivery teams had a variety of experience across health and social care in the charitable sector and statutory organisations. Three staff were full-time and four staff were part-time. One member of staff left before the end of the project to go on maternity leave. Additional information relating to the composition of the sampled clusters can be found in Table 2.

We observed thirteen delivery team training sessions. All staff, except one team leader, attended all training sessions. We also interviewed the seven delivery team staff: PICs (*n* = 3); support workers (*n* = 2) and team leaders (*n* = 2). These interviews were a combination of post-training interviews, interviews following a two-month review or graduation of older people and exit interviews at the end of the intervention. See Table 3.

We interviewed twenty of the twenty four older people who had been offered the intervention and recruited to the process evaluation across the four clusters. A carer was present and contributed to the discussion during two of these interviews. The characteristics of participants in the sample is shown in Table 4.

**Table 4:**
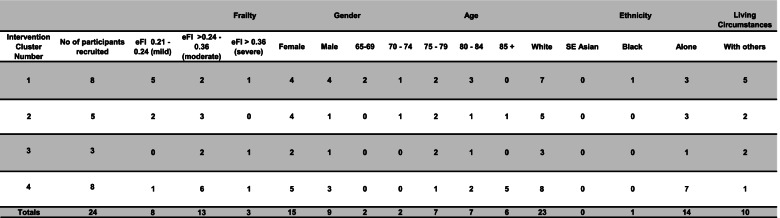
Process evaluation participant characteristics

Key: efi cut points based on eligibility criteria (0.21 and above), and categories of mild, moderate and severe (Clegg et al., 2016). Age range cut points-cut oint at 69/70 based on Windle et al. (2009) shift in resilience evident between 60–69 category and 70 + ; cut point of 85 “oldest old”

Of those older people interviewed, thirteen had already participated in the observations. The remaining seven individuals had declined or withdrawn from the intervention (referred to as limited/non-engagers) for a variety of reasons as explained in the findings. Our sample comprised mostly White British females. Just over half were living alone and had moderate frailty. Six were over the age of eighty five.

We interviewed five primary care staff, three GPs and two practice managers who provided insights into primary care integration of PROSPER.

Three key themes relating to the implementation of PROSPER emerged: a mismatch between training offered and the realities of the target population; a lack of integration of delivery teams into primary care and the complexities of engaging older people in PCP. These are reported here.

### Mismatch between training offered and the realities of the target population

#### Delivery teams had to adapt their role to meet the realities of the intervention

Although members of the delivery team retained key messages from training, they felt inadequately prepared to deliver PROSPER in practice, particularly working with older people earlier in the frailty trajectory. This was observed during delivery of the intervention and reported in exit interviews with the delivery team. The original Age UK National training, which was drawn on heavily in the feasibility trial, neglected to mention the preventative aspect of PCP for older people with moderate frailty and instead emphasised having an immediate beneficial impact. Therefore, the PROSPER training focused on facilitating transformative change rather than preventing transition to a worse frailty state. This meant that delivery teams anticipated older people already experiencing significant effects of frailty presenting with clearly defined needs. Although the intervention was targeted across the frailty spectrum mild–severe, the distribution of eFI scores for all participants including those receiving the intervention was skewed to the lower end of the frailty spectrum. Therefore, participants were more physically robust, better socially connected and less open to transformative change than expected,

“They’re very socially connected and well supported compared to what I was conjuring up in my mind and seemingly less frail. I was expecting them to perhaps be a little bit further along the trajectory” (PIC 3, end of intervention interview)

Despite coming from different backgrounds, on the whole, delivery teams successfully acquired skills and knowledge and retained specific shared key messages.

Observations demonstrated that delivery teams engaged well with training despite initially struggling with the ‘guided conversation’ and motivational interviewing sessions during training as this was largely a new way of working for the majority of the delivery team staff. Access to trainers with effective coaching skills and the opportunity to practise techniques in a supportive environment were important in skill acquisition and maintenance. Post training delivery teams agreed on key messages, i.e. a person-centred approach, enabling older people to lead in decision-making and to ‘facilitate not fix’,

“I would have gone in before and said, ‘right this is what I’m going to do for you’. I fully understand that it’s my job to get them to come up with what their goal is, what they want from life and my role is to guide them to get to that" (PIC 1, end of intervention interview)

How delivery team staff enacted the training was observed in ‘guided conversations’ (*n* = 17), two month reviews (*n* = 2) and ‘graduations’ (*n* = 7).

### The content of ‘guided conversation’ training sessions lacked clarity

An over-reliance on Age UK National training, which was delivered by trainers unconnected to the development of the intervention and with limited input from the PROSPER team, had negative consequences. Observed ‘guided conversation’ sessions lacked structure, included irrelevant content and terminology sometimes lacked a clear explanation. The process of initially engaging with older people was ill-defined with an over emphasis on the ‘guided conversation’ component of PCP at the expense of the wider process, i.e. goal setting, action planning and reviewing. Consequently, it was clear from post-training interviews that whilst delivery teams grasped the basic concepts underlying PCP, such as encouraging the older person to take the lead throughout the process and facilitating outcomes rather than providing solutions, they struggled with understanding how the ‘guided conversation’ should be operationalised within the context of the PROSPER service. Delivery teams also recognised the tension inherent in a process that is supposedly led by the older person’s perception of their needs, but also covers specific pre-defined topics,

“Although we’ve got some themes that we’re going to work through each time, it’s not going to be done in a certain order on a certain checklist, so gathering the information and being able to let the conversation go as the person wishes without an agenda” (PIC 3, end of intervention interview)

#### Training overall lacked cohesion and a defined PROSPER identity

Delivery teams appeared to understand the content of individual training sessions and demonstrated that they had acquired knowledge and skills. However, they had difficulty applying these attributes across the training package as a whole. For example, delivery teams were observed to have mastered and reported that they understood behaviour change techniques, but observations indicated that these were not successfully integrated into other elements of the training and delivery teams reported that they were unclear how behaviour change techniques should be used in the context of PROSPER,

“We did use a case study to try and look at that (BCTs), but I did wonder whether it would be possible to, be integrated into one of the other sessions in some way to make sense of it” (PIC 3, end of intervention interview)

#### Timing of initial training and top-up is important

Delivery teams reported that the lengthy gap between their training and intervention delivery had an adverse effect on morale and recall,

“Generally the training you know for future reference can probably be rejigged and compressed within a timeframe so that it’s actually closer to that commencing to delivery, just so that it’s a little bit fresher in your mind, it’s amazing how quickly things can slip from the mind” (PIC 3, end of intervention interview)

Also, delivery teams would welcome an opportunity to revisit the training and review their practice as part of the delivery process,

“I think that the training was excellent, but it was very front-loaded and it would be really useful to be able to kind of have review points through the process, through the system or to set up some good practice forums, or something like that, to kind of keep reminding ourselves” (PIC 2, end of intervention interview)

#### Training provided insufficient support in operationalising the intervention

Delivery teams reported a lack of guidance and support in delivering practical aspects of the intervention during the initial delivery period, e.g. the completion of required documentation and the timing of reviews and graduations. Less experienced staff said they wanted regular contact with trainers and the research team during the delivery of the intervention in order to discuss their cases,

“I feel like we should have been supported when we had our first two or three referrals …somebody in the team checking how have you gone on with that person, doing the paperwork and putting it on to [database] in the right way” (Support worker, end of intervention interview)

Team leaders in Age UK were not always equipped to support the delivery teams (PICs and support workers) effectively due to absence at the training sessions. This resulted in knowledge gaps and a lack of detailed awareness of the intervention requirements. The more experienced team leader had prior knowledge of PCP and supported the PICs and support workers through frequent supervisory meetings.

### A lack of integration of delivery teams into primary care

#### Relationships between the delivery teams and primary care staff were tenuous

Integration between delivery teams and primary care staff did not happen as intended. All of the delivery team staff, even those who had a background in health or social care and had prior experience of working in a clinical setting, struggled to develop a strong relationship with the primary care staff in any of the participating practices. This was due to a lack of confidence and uncertainty around what to expect in the practices and uncertainty around what primary care staff expected of them. Delivery teams soon stopped attending MDT meetings on a regular basis reporting that they were not routinely invited; they felt unwelcome and undervalued. Consequently, staff found themselves disconnected from the practices they were working in,

“It feels very much like we’re just working in isolation and it wasn’t supposed to be like that. But it really does feel very isolated” (PIC 2, end of intervention interview)

Difficulties in building effective relationships were sometimes exacerbated by the contrasting cultures of the charitable sector and primary care sectors. The absence of a well-defined collaborative relationship meant that the delivery teams had difficulty accessing the physical resources, e.g. computers and printers, in the practices. This resulted in inefficient working practices with delivery staff returning to offices or their homes to complete administrative tasks remotely.

#### Primary care staff did not ‘buy-in’ to the intervention and attached limited value to their involvement

Delivery teams perceived that primary care staff generally had a limited understanding of PROSPER, including their own role and that of the delivery teams which meant there was a lack of engagement,

“I don’t think the surgeries have any ownership of the project that’s going on; it’s just been kind of landed on them. They’re doing the bare minimum that they have to. We’re kind of working with the little bits of information they’re giving us, but they’re not actually involved or seeing the results of what we’re doing. So, there’s nothing in it for them, is there?” (PIC 2, end of intervention interview)

Delivery teams stated that primary care staff were reluctant to share information with them which meant there was no discussion relating to the previous management of older people and their potential needs before the delivery teams visited older people. Consequently, delivery teams often felt ill-prepared. Following their visits, delivery teams were reluctant to give feedback to the primary care staff because most of the older people they encountered had limited or non-medical needs,

“I haven’t felt there was anyone in particular to take along to their MDT meetings. I think they might have wondered ‘Why are you coming to talk to us about somebody who wants to apply for a Blue Badge or Attendance Allowance?’” (PIC 2, end of intervention interview)

Conversely, primary care staff perceived that there had been a lack of feedback on the part of the delivery teams, but reported that they would have welcomed the sharing of information,

“Even if it was every three or six months someone came and said, ‘Right, we’ve had four patients in this time period and out of these four patients they were A-B-C-D and this is what we’ve done’” (Primary care staff, end of intervention interview)

Primary care staff valued their involvement in PROSPER in respect of its contribution to improved service and patient care and the Care Quality Commission inspection. In addition, they appreciated the opportunity to utilise the delivery teams to support existing members of staff, for example, occupational therapists and community matrons.

### The complexities of engaging older people in PCP

#### Intervention uptake was influenced by individual circumstances

Older people who took up the intervention reported feeling listened to and valued the objective viewpoint of the delivery teams as opposed to the subjective perspective of family members and carers,

“I’ve really enjoyed talking to my PIC about how I’m feeling, how I fit in with the household and what I do here” (Older person, graduation interview)

In addition, they appreciated the delivery teams’ knowledge of services and the support they provided in facilitating the development of self-management skills,

“It was really useful to me and if I need anything in the future I know that Age UK is a phone call away and I’m sure they would help me” (Older person, graduation interview)

They referred to a range of benefits including an increased awareness of local services, support with accessing practical and financial assistance and increased confidence and motivation to address specific issues in their lives,

“It’s (PROSPER) helped me stop feeling sorry for myself and get up and get cracking. That sort of thing” (Older person, graduation interview)

Intervention uptake by older people was influenced by timing, with those in the midst of life changes such as moving to a new area, engaging particularly well. Others felt they had benefitted from the intervention even when well-supported by family and friends. Delivery teams reported that non-engagers also viewed the intervention positively, albeit for a different target population, i.e. those living alone and/or with readily identifiable health or social needs,

“…the common comments that I get from people I see is we think it’s a great service but we don’t need it, I’m sure you’re needed more elsewhere. We think it will be better for somebody who’s living by themselves or whatever” (PIC 2, end of intervention interview)

#### Older people lacked an understanding of the purposes of the intervention and how it could be useful to them

Prior to the first PIC visit, older people received an information sheet and an introductory telephone call from a PIC, who introduced PROSPER. Both the observations and interviews highlighted that, at the time of their first visit, older people often lacked a full understanding of the intervention’s intended purposes, specifically to improve quality of life and prolong independence. Delivery teams also reported that older people often did not know why they had been offered the intervention, with younger individuals (65–75) in particular questioning why they had been targeted. Older people reported a range of expectations before the initial visit; some assumed they would be examined by their doctor and have medical needs addressed or travel somewhere for a physical examination,

“I thought they’d probably begin by examining my health you know. I thought they’ll be using me as a fitness test for instance and then comparing it with other people” (limited engager, older person interview)

Others expected a survey focusing on local older people’s needs or a project to improve practice-based services for older people. Even after their initial face-to-face meeting with their PIC many older people appeared to have difficulty understanding the purposes of PROSPER. PICs reported that this lack of understanding was partly due to older people not being familiar with a “community intervention” approach. One PIC commented,

“I think some of them, even after the ’guided conversation’ and even with my best efforts, still don’t really fully understand what it’s about” (PIC 3, end of intervention interview)

Observations suggest that PICs were inconsistent when describing the intervention, the older person’s role in it and how they may benefit. On occasions, PICs provided no explanation and sometimes it differed between the individual PICs.

#### Some older people had difficulty with goal setting and action planning despite PICs’ attempts to problem-solve collaboratively

Despite PICs clearly demonstrating the ability to build rapport and identify areas of importance through the motivational interviewing techniques (open questions, reflections and summaries) they had learnt, observations demonstrated that many older people struggled to identify goals. PICs also reported that older people found it particularly difficult to imagine a future state that they could aspire to,

“I say ‘I want you to try and think what you would like to be like in six months’ time. How do you see yourself?’ Apart from a few that have said ‘Dead!’ they say ‘Hopefully I’ll still be here, love’” (PIC 1, end of intervention interview)

Difficulties in identifying goals and deciding that the intervention lacked relevance for them resulted in around a third of participants not engaging with the intervention. Older people reported that they did not feel in need of it,

“We both decided that neither my wife nor I needed it. We’re financially secure, we’re both fit as butcher’s dogs,” (limited engager, older person interview)

Monitoring data showed just less than two-thirds of the participants across the study developed an action plan, with similar rates across the two localities. Around three-quarters of participants set one or two goals, but up to nine goals were recorded on occasions. When goals were agreed, observation and interview data showed that they were often identified following the PIC introducing topics (e.g. health and fitness, transport and mobility, medication) by rote rather than as part of a natural conversation. Monitoring data showed common goals included benefit checks, applications for parking permits and information about transport and community based social groups. Goals were rarely explicitly associated with behaviour change or empowering older people to avoid transitioning to a state of increased frailty and dependency. Approximately, two thirds of ‘goals’ recorded in the monitoring data could be considered ‘enablers’, i.e. a means to an end, not the end point in themselves. PICs reported that collaborative problem-solving and action planning was often unsuccessful because older people assumed it was the PICs role to take the lead and older people lacked the necessary knowledge of services available in the community. Observations demonstrated that agreed actions were largely the remit of PICs, rather than older people completing them independently or with support. Many appeared to be a reaction to the older people’s immediate need, e.g. providing information. PICs thought that it was easy to offer suggestions or actions unconsciously and acknowledged they should have ‘allowed’ the older person to think more for themselves. One PIC commented on the ease with which they could start identifying solutions,

“There would be times when you would sort of make a suggestion and then be conscious that that might be putting something on a plate in front of somebody” (PIC 3, end of intervention interview).

Furthermore, PICs felt an artificial pressure to achieve something during the interaction to comply with the trial monitoring requirements,

“On the paperwork, [no dots here] it’s goals, actions, has the goal been achieved, yes or no? And, you know, you feel like you’ve failed if you’ve ticked no”. (PIC 2, end of intervention interview).

Goals that were successfully identified by participants and delivery teams were recorded as achieved in the majority of cases. Where goals were recorded as unachieved the reasons were: participant illness; participant disengagement and third party issues.

## Discussion

Our process evaluation has highlighted important lessons on the implementation of PCP for people with frailty. First, despite observed engagement in the training sessions and retention of key training messages, delivery teams felt underprepared for working with people with lower levels of frailty. Second, delivery teams were not integrated into primary care in the way that was expected, which impacted on the resources they had at their disposal, led to some inefficient working practices and limited information exchange. Third, engaging older people in PCP was complex and shaped by individual circumstances, their understanding of the service and potential relevance for them and difficulties with identifying goals and action planning.

### Delivery team training

Overall, training was positively received. However, we identified managing expectations and perceived outcomes as areas that were problematic for the PICs, all of whom anticipated working with older people with greater frailty and need. This was likely to have been exacerbated by the reliance on trainers from the National Age UK programme which had a different, more vulnerable, target population. Evidence is lacking relating to training mismatch and practice in the field of health and social sciences. However, literature on training and employee performance suggests that a mismatch is associated with feelings of unmet expectations amongst employees and ultimately, worse outcomes in terms of job productivity [21]. Unmet expectations were highlighted in the findings and may have impacted on how PICs judged the success of the intervention, how rewarding they found their role and how they demonstrated the value of the intervention, which may have led to ‘weak’ engagement as discussed below.

The provision of support for the PICs, both during training and post-training was important. Evidence suggests that a key aspect of training programmes is the support that is available through reinforced learning, primarily in the form of opportunities to rehearse and receive feedback [22]. In addition, continuing support following initial training is important in maintaining successful intervention delivery [23]. Limited ongoing support for the delivery of PROSPER was provided by the trainers and researchers and, to a lesser extent, the team leaders. Team leader engagement in the training was minimal and a subsequent lack of knowledge of PROSPER and its delivery impacted on the support that they were able to provide the PICs. As exemplified by the literature, time constraints and competing demands from other projects were both highlighted as factors explaining why individuals may not have received the support they needed from their team leader [24].

### Working with the primary care staff

Although a range of approaches were used to engage with the cluster practices, our results show that integration between the primary care staff and the delivery teams did not fully take place. This was even the case for those PIC workers who had previously worked in healthcare. This was mainly due to issues with communication, understanding of the intervention and differences in organisational culture which impacted on individuals’ and primary care staff’s expectations and responsibilities. Consequently, primary care staff were generally unaware of the intervention and the potential benefits for their older patients.

These findings are consistent with research which suggests that cohesiveness in terms of working relationships between different parties is essential for successful implementation, in particular complex interventions [25]. Furthermore, stakeholders’ perceptions of the benefits afforded them, including statutory drivers, by engaging with interventions is crucial in determining their support and buy-in [26, 27]. If stakeholders lack a full understanding of what the intervention entails and have other competing demands on their time [23, 28] facilitation of the intervention is likely to be compromised [29].

### Intervention uptake

Despite receiving an information sheet and an introductory telephone call from a PIC, many older people lacked an understanding of the purpose of the intervention. Due to the trial design, a gap of several weeks sometimes occurred between older people receiving their information sheet and the initial visit. This may explain why older people did not fully understand the purpose of the intervention. This lack of recall is corroborated in the literature [29]. Older people’s lack of understanding sometimes persisted throughout the duration of the intervention. This may be as a consequence of the mismatch between PIC’s rehearsed explanations developed in training, which were intended for older people presenting with clearly identifiable unmet needs, and the reality of the older people that the PICs encountered, many of whom reported few current difficulties. This was likely as a consequence of the original training being grounded in the original Age UK programme, which had a slightly different target population and recruitment pathway.

Identifying potential goals was challenging for older people. In the literature, the characteristics of individuals that struggle with problem solving have been cited. These include difficulties grasping the concept of goal setting or viewing it as a selfish activity, acceptance of their situation and current stressors such as ill-health predominating thoughts of the future [30], all of which are more likely in an older frailer population. It is possible that PICs were insufficiently prepared to help older people overcome these barriers to goal setting, due to the facilitation of the motivational interviewing component in training which was delivered as a group. Although this prepared them for *identifying* significant areas of importance for older people, in terms of *working with* older people, group training activities may have provided less effective grounding. The literature suggests that individualised coaching over time, based on interactions with older people rather than workshops alone, is necessary for individuals to develop skills in motivational interviewing [31]*.* Although this has significant cost implications in terms of personnel required for training, this needs to be weighed against the potential negative impact on intervention delivery, specifically the delivery of a training programme that does not fully meet the needs of recipients.

When goals were identified, both older people and PICs often found shared decision-making and action planning difficult. Shared decision-making is recognised as challenging for those implementing it [32]. Indeed, a review on shared decision-making approaches with older people suggests that although older people would like to be involved in decision-making it is unfamiliar to them and they are often not encouraged or enabled to do so. Reasons cited for this include limited time, power imbalances and practitioners’ biomedical focus [33]. Achieving collaborative approaches is dependent on relationship building and enhanced communication skills.

### Practice implications

Our findings have implications for both our own definitive trial and wider PCP implementation. We recommend that delivery teams have training tailored to their target population, opportunities for skills refreshment (via access to trainers, on-line materials or practise) and on-going support mechanisms. The content and timing of the PROSPER training for the definitive trial was revised based on feasibility feedback. In addition, on-line training and on-going access to materials was introduced due to the necessity to deliver at the start of the definitive trial when pandemic restrictions were still in place. We also recommend that primary care staff are made aware at the start, the level of engagement in PROSPER and their support for delivery teams that is expected from them, including a named clinical ‘champion’ and ‘admin buddy’ to work with the delivery teams. In addition, delivery teams need to make strenuous efforts to meet with primary care staff as soon as possible to explain their role, answer questions and agree appropriate feedback mechanisms for individual practices, e.g. case studies or meeting attendance. Finally, we have developed a standardised video to introduce the intervention to older people. The effectiveness of this approach is being tested in a study within a trial [34].

### Strengths and limitations

This is the first time, to our knowledge, that the use of PCP for older people with frailty has been studied. The process evaluation used a range of different data collection methods to explore context, implementation of the core intervention components and stakeholder experiences from a range of stakeholders including limited/non-engagers and those with severe frailty.

The strengths of the process evaluation were that we conducted observations of training and at key points during the delivery of PCP and gathered the views of older people and carers, alongside delivery staff and members of primary care staff across the four clusters. In addition, we were able to substantiate our findings with pertinent monitoring data collected as part of the feasibility trial (to be fully reported elsewhere). Integrating multiple types of data can be challenging. Iterative data collection and analysis facilitated this process, for example our analytic summaries of observations of the training sessions were used to inform the topics discussed in the interviews. This enabled us to build an in-depth understanding of implementation processes, allowing us to bring together our analysis of the observations and multiple interviews (for example, despite good observed engagement in the training and grasp of key messages, in exit interviews PIC workers reported that they were not sufficiently prepared to work with older people with lower frailty scores).

The weaknesses for the process evaluation were largely a result of trial procedures or constraints. For example, our sample lacked ethnic diversity, reflecting wider trial participation. In addition, the process evaluation (and intervention delivery period) was truncated from twelve to nine months because of the trial randomisation process, difficulties in securing delivery team funding in one locality and the imperative to conclude before the funding deadline in March 2020.The lack of engagement with primary care staff also meant that the research team were unable to observe and attend MDT meetings as intended. During the first quarter of 2020 there were still eleven participants in receipt of the intervention. However, as the UK (and other countries) prepared for ‘lockdowns’ during the COVID19 pandemic, service delivery was reduced to telephone contacts, in some cases curtailed altogether. However, we were not able to explore this as access to participants and PICs was severely restricted.

## Conclusion

The experience of individual older people reported here suggests that PCP is a promising approach to address the individualised needs of older people with frailty, extending beyond the traditional medical model of care to a more socially orientated approach that is required for this group. Careful tailoring of the training is necessary to enable delivery teams to deliver PCP to older people with frailty, who are likely to have a broad range of personal circumstances and priorities. Ideally, supportive on-going individual training should be available. Considerable efforts are required to integrate delivery teams, who have the background and skillset required for PCP for older people with frailty, into the healthcare team and who fully understand the expectations and contributions of each agency from the outset. In addition, the target population of older people with frailty will need time and support to adjust to new ways of thinking about their own health now and in the future, aligned with the shared decision making approach that is central to PCP. Addressing these key factors will be essential in our definitive trial and when developing future models of care for delivering PCP to support older people with frailty to sustain their independence and quality of life.

## Supplementary Information

**Additional file 1: Observation Template.** Process Evaluation of Personalised Care Planning (PCP) for Older Adults (OA) with Frailty.

**Additional file 2: Topic Guide 1.** Age UK PIC / SW Post-Training Interview.

**Additional file 3: Topic Guide 2.** Age UK PIC Two Month Review Interview.

**Additional file 4: Topic Guide 3.** Age UK Team Leader Exit Interview.

**Additional file 5: Topic Guide 4.** Age UK PIC Exit Interview.

**Additional file 6: Topic Guide 5.** Age UK SW Exit Intervie.

**Additional file 7: Topic Guide 6.** Older People “Graduation” Interview.

**Additional file 8: Topic Guide 7.** Older People “Limited Engager” Interview.

**Additional file 9: Topic Guide 8.** PCT Interview.

**Additional file 10:** Personal Independence Co-Ordinator Job Descripton.

## Data Availability

All data generated or analysed during this study are available from the corresponding author on reasonable request.

## Abbreviations

BCT: Behaviour Change Technique
eFI: Electronic Frailty Index
GP: General Practitioner
MLTC: Multiple Long Term Condition
MDT: Multi-Disciplinary Team
NHS: National Health Service
PCP: Personalised Care Planning
PIC: Personal Independence Coordinator
PROSPER: Personalised Care Planning for Older People
RCT: Randomised Control Trial
