# Supplementary material for: Implementing personalised care planning for older people with frailty: a process evaluation of the PROSPER feasibility trial
Source: BMC Geriatr. 2022 Sep 16;22:760. doi: 10.1186/s12877-022-03426-4 (PMC9479257; doi:10.1186/s12877-022-03426-4)
Supplement: Supplementary file 1 — Additional file 1: Observation Template. Process Evaluation of Personalised Care Planning (PCP) for Older Adults (OA) with Frailty. [file 12877_2022_3426_MOESM1_ESM.docx]

**Observation Template: Process Evaluation of Personalised Care Planning (PCP) for Older Adults (OA) with Frailty.**

In accordance with the iterative nature of qualitative research (such as observation of interaction and related events), this template may be refined drawing on on-going data collection and analysis. This document intends to offer guidance to the researcher observing interactions in formal and informal situations, rather than constraining the generation of data.

The following template prioritises 9 areas of observational focus. Each of these 9 topics form a framework that was devised by Spradley (1980) in his book *Participant Observation*.

| Date |  |
| --- | --- |
| Location  (Training venue, OA home etc) |  |
| Observation Number (for researcher fieldnotes) |  |
| Aims and learning outcomes of training session / PCP in OA home observed |  |
| Duration of observation |  |
| **Focus of observation**:  Spradley’s framework of participant observation  (consisting of 9 areas of observational focus – see right).  Spradley, J.A. 1980, *Participant Observation*. Belmont, Wadsworth Pub. | These are general observations, to capture a sense of how the process of the training, guided conversations (GC) and MDTs went in practice.  This 9-point guide (Spradley 1980) assists the researcher in areas to observe during interaction.   1. Description of the social and physical **space** where the training/PCP took place (including built environment, furnishings, how training/PCP was organised within space, how trainers/PIC used space effectively and safely, how are MDTs arranged) 2. Capture a sense of the **actors** in attendance (who/what else is present in the room - friends, relatives, pets other health professionals, particularly in MDTs are the GPs there?; what influence do they have upon the interaction) 3. Description of what **activities** take place during the training/GC visit – assessments, reminiscence, eating, drinking, health consultations, other conversations. For guided conversation, pay attention to the topics covered, motivational interviewing techniques and the BCTs used. 4. A description of the **objects** used during training/ GC (i.e. how presented to staff, approach to learning and teaching adopted, opportunities for learner participation, organisation and structure of session, delivery and pace, resources used, exercises used, how learning was assessed, clarity of what was communicated). 5. Description of **acts** between trainers and staff and OA (with regard to who is doing what?) Description of interactions between those undertaking the each act (who guides, who leads, who decides, who refuses). Note how actors are included or excluded particularly in MDTs – do the PICs have to ‘fight’ to be heard? 6. Description of **events** that were not planned (fire alarms, visitors, telephone calls, pets: do they disrupt or facilitate the interaction) 7. Description of **time** and timings: how long did observation last? What time is spent assessing? What time is spent talking more generally about non project topics? Was time sufficient / insufficient to complete intended goals/targets. How much time is allocated to PICS in MDTs? 8. Describe **feelings** and emotions that were observed during the interaction (were participants upset, happy, interested, disinterested, embarrassed, enthusiastic, angry, ambivalent, motivated). Do moods and emotions shift during the assessment? Do any problems arise from other actors present? 9. Describe the close of the meeting in terms of **goals**, goal achieved and goals agreed (for next session/visit)Include descriptions of informal / unplanned activity that appeared influence the delivery of the training / GC sessions   Researcher may add notes relating to social cognitive theory, social theory etc. Anything of academic interest that may have been observed in the session. |
| Open fieldwork notes  *Detailed description of the event observed.* |  |
| Possible points / issues to follow up in interviews |  |
| Reflective comments | Ethnographic reflexivity may be attached here. |
