# Supplementary material for: Implementing personalised care planning for older people with frailty: a process evaluation of the PROSPER feasibility trial
Source: BMC Geriatr. 2022 Sep 16;22:760. doi: 10.1186/s12877-022-03426-4 (PMC9479257; doi:10.1186/s12877-022-03426-4)
Supplement: Supplementary file 2 — Additional file 2: Topic Guide 1. Age UK PIC / SW Post-Training Interview. [file 12877_2022_3426_MOESM2_ESM.docx]

# Topic Guide 1: Age UK PIC / SW Post-Training Interview

**General prompts**

Could you tell me a bit more about ….

Could you give me an example of when ….

How did that make you feel?

Has that changed over time?

*When introducing the interview, reiterate that the training we*

*are discussing in the interview is the PROSPER training*

*(GC, MI, BCT, Mental health, frailty, research process, role play)*

### Opening questions

Could you tell me what attracted you to the role of PIC / SW?

Could you tell me what you thought about personalised care planning prior to taking on this role?

- Had experience of doing PCP/training on PCP?
- How knowledgeable/confident did you feel about it?

### PROSPER training

What were you expecting from the PROSPER training?

- How did you feel about attending the training?
- Did you receive any information about the training in advance?
- Did you feel the training was delivered in the appropriate order?

How did you find the training?

- What comments do you have about the training being delivered online?
- Did you learn new information/skills (prompt for key skills)
- Were there any issues covered in the training that were particularly important/useful for you? (GC, MI, BCT, mental health, frailty, research process, role play?).
- Were there any aspects of the training that you felt were more or less relevant to your particular role
- Were there any aspects of the training you found easier/harder to engage with than others? Why?
- Did you think the training helped you understand how you could apply any new information/skills in practice?
- What did you think of the materials provided by the trainers? Have you referred back to them at all?
- Do you feel the training provided supports the material provided in the intervention manual? Have you got any unanswered questions about the ‘fit’ between the manual and what was covered in the training?
- Are there any questions you would have liked to have asked the trainers?
- Have you followed up on any aspect of the training yourself, e.g. more research? What? Where?
- Is there anything that helped/hindered you in getting the most out of the training?

What did you gain from the training sessions?

- Awareness, knowledge, skills, confidence, preparedness?
- How important do you feel this is?
- Could you give me an example?

Do you feel the training helped clarify your role?

Is there anything the training did not cover that you think would have been helpful?

Are there any key skills or knowledge from the training that you will take forward into your practice?

- Prompt for GC, MI, BCTs.
- How do you think you’ll apply this?
- How would that be different to what you would have done before the training?

Are there any techniques or information covered in training that you feel would be difficult to apply in practice?

- Prompt for GC, MI, BCTs.
- Why?
- Anything that would help to better prepare you?

How did you find the ‘your team and your role in the wider team’ virtual training session?

- Did you find the material relevant for your role?
- Was this an appropriate way of delivering this material? Could you suggest any improvements?
- Would delivering training in this way (workbook followed by a virtual session) be appropriate for any of the other material covered in the training?
- Have you undertaken any other training virtually / on-line (i.e. mental health training)? How did you find this?

Are there any other changes you would make to the content or delivery of the training?

- Content, delivery style, order of sessions, materials provided
- Has the training been timely in relation to the start of service delivery – too long/short

Do you feel it was helpful for your supervisors to attend the training?

- If yes, any particular sessions?
- Why?

Did the training help shape the team dynamics in any way?

- Did it enable you to come together as a team? In what way?

## Closing questions

On a scale of 1-10 (very prepared) how prepared do you feel about delivering the PROSPER service? What, if anything, do you think could improve your preparedness?

Would you recommend any elements of the PROSPER training for people undertaking PCP with older people? Why/why not?

Is there anything else you think I need to know to understand about the PROSPER training and how it may influence your future practice?

Any questions?
