# Supplementary material for: Implementing personalised care planning for older people with frailty: a process evaluation of the PROSPER feasibility trial
Source: BMC Geriatr. 2022 Sep 16;22:760. doi: 10.1186/s12877-022-03426-4 (PMC9479257; doi:10.1186/s12877-022-03426-4)
Supplement: Supplementary file 3 — Additional file 3: Topic Guide 2. Age UK PIC Two Month Review Interview. [file 12877_2022_3426_MOESM3_ESM.docx]

**Topic Guide 2: Age UK PIC Two Month Review Interview**

(NB: The Feasibility Study protocol states that the two month review point observations and interviews are intended to record **the way that the action plan is negotiated/implemented in practice** and **to explore the facilitators and barriers to implementation**)

*When introducing the interview, reiterate that the questions relate to the older person (OP) who has just been observed to have received their 2 month follow up*

**Opening questions; general impressions of how *the two month review* went**

- How do you feel today’s review went?
- Were there any surprises or was it as you expected?
- What do you feel were the major successes today?
- Did you encounter any significant problems today? What were they?

**Questions about working with this specific OP (and their significant other(s)) *over the last 2 months***

- To what extent do you think this OP has understood the underlying aims and purposes of PROSPER?
- Why do you think this OP initially agreed to receive the intervention?
- Why did they agree to participate beyond the first meeting?
- To what degree did you feel you built rapport with the OP (can you tell me what helped/hindered relationship building)?
- To what extent did the OPs significant others engage in the PCP process (if applicable)?
- Have you felt that you have had adequate knowledge and skills to work with this individual effectively (if not what would have helped)?
- What practical issues have you encountered when providing PCP to this individual (for example transport, communication or environmental difficulties)?

**Questions about the guided conversation, identifying goals and action planning with this OP *during the last two months***

- To what extent was the OP able to take an active role in identifying the issues/goals they wanted to work on?
- What role did the OP take in problem solving/action planning?
- To what extent were the identified goals realistic?
- What BCTs were you aware of employing during the PCP process?
- What MI techniques did you employ during the PCP process?
- What key skills have helped you to deliver PCP to this individual?
- Can you think of anything which could have made this process more successful?

**Questions about actions undertaken as a result of this OP receiving PCP**

- What actions were undertaken as a result of this OP receiving PCP?
- What barriers and facilitators influenced the degree to which these actions were successful?
- Are there additional actions (by the OP or others) that you think may enhance their wellbeing?

**SW and other Age UK staff involvement**

- Was the SW (Liz/Amy) involved with this OP? If yes, can you tell me more about this?
- What factors influenced the success of their involvement (e.g. capacity, knowledge of services, transport issues)?

**Support from AGE UK (supervision, etc.)**

- When working with this OP have you felt that you have had sufficient support from Age UK both in the form of supervision for you or in terms of service provision?

**Working with OPs GP/wider MDT**

- How has your connection with GP practice and wider MDT influenced how you have delivered PCP to this OP and its subsequent impact?

**Referring onto community services**

- Did you undertake any referrals onto community services? If so, how did you find this process?
- How could community services be improved?

**General impressions of how PCP is working for this person**

- To what extent do you feel PCP has helped this OP?
- What difference has it made to their wellbeing?

Close
