# Supplementary material for: Implementing personalised care planning for older people with frailty: a process evaluation of the PROSPER feasibility trial
Source: BMC Geriatr. 2022 Sep 16;22:760. doi: 10.1186/s12877-022-03426-4 (PMC9479257; doi:10.1186/s12877-022-03426-4)
Supplement: Supplementary file 4 — Additional file 4: Topic Guide 3. Age UK Team Leader Exit Interview. [file 12877_2022_3426_MOESM4_ESM.docx]

**Topic Guide 3: Age UK Team Leader Exit Interview**

*Opening*

- *What are your general impressions of how the PROSPER service as a whole has been working?*
- *Can you tell me about your role in PROSPER?*
- *Describe your relationship with the delivery team?*
- *How have you found taking on the role of team leader?*

*Training*

- *What are your views on team leaders attending the delivery team training? (do you think they have had sufficient training? Why do you think that?)*
- *What would you say was the extent of your knowledge of the PROSPER service?*
- *What could be done to help you in terms of training/knowledge of the service?*

*Overall impressions of how service is working*

- *What if anything has surprised you during the delivery phase?*
- *Can you talk about any barriers/facilitators to implementation of service?*
- *What, if anything, can you think of that would improve the service?*
- *What has been the extent of your role in establishing links between the DT and MDT/GP practice?*
  - *Do you have a view on info sharing and communication with MDT?*
- *What are your thoughts on communication between yourself and the DT?*
- *What are your views on the type of OP taking up the PROSPER service?*
- *It has been suggested that the service has been targeting the wrong older adults, what are your views on this?*

*AGE UK team*

- *How have you found managing the DT alongside your wider Age UK remit?*

*(Managing case load, Resources and infrastructure)*

- *How are PICs/SW supported (supervision, pastoral, advice on tricky cases)*

*• Any local/national issues impacted on the service?*

- *How well do you think the members of the DT have been working together?*
- *Do you have a view on how effectively the support worker is integrated within the DT?*

*Research*

- *How have you found your interactions with the research* *team (AH, CTRU)*

*Local/national context*
