# Supplementary material for: Implementing personalised care planning for older people with frailty: a process evaluation of the PROSPER feasibility trial
Source: BMC Geriatr. 2022 Sep 16;22:760. doi: 10.1186/s12877-022-03426-4 (PMC9479257; doi:10.1186/s12877-022-03426-4)
Supplement: Supplementary file 5 — Additional file 5: Topic Guide 4. Age UK PIC Exit Interview. [file 12877_2022_3426_MOESM5_ESM.docx]

**Topic Guide 4: Age UK PIC Exit Interview**

*When introducing the interview state that the questions are going to relate to the PICs overall experience of delivering the intervention throughout the delivery phase*

**Opening Questions; *general* impressions of how the PROSPER service has gone**

- What are your general impressions of how the PROSPER service as a whole has been working?
- What if anything has surprised you during the delivery phase?
- Can you talk about any barriers to implementation of service?
- What has helped with service implementation?
- What are your views on the OP that have been taking up PROSPER? (Do you feel that it has been reaching the right people and if not what could be done to target PROSPER more effectively?)
- What, if anything, can you think of that would improve the service?

**The process of delivering the Service**

**Your role**

- How have you found taking on the PIC role?
- What were you expecting from the PIC role?
- What has it been like in reality?
- Has your role evolved during the course of delivering PROSPER, if so how?

**Organising the first visit**

- How did you find the system for identifying OP interested in taking up the service and alerting you to them (did you feel that you were able to access sufficient information about the OP through links with MDT (SystmOne, etc.) before the first visit)?
- What were the pros and cons of the system?
- What has been your experience of establishing the initial contact with OP and arranging visits?

**Delivering PCP to OP**

- How did you find the process of introducing the PROSPER service during the first visit?
- To what extent do you think the OP generally “got” it?
- Can you tell me about what elements of your training (i.e. GC, MI, Frailty, BCT) you found yourself employing (or not)? Can you talk a bit more about why things worked out this way?
- To what extent were goals identified during the GC process?
- Can you tell me about the process of action planning (I.e. to what extent was it collaborative)?
- What issues did you face implementing the action plan?
- How did you find the process of referring to services?
- To what extent have your skills in delivering PCP developed over time? (If they have developed what are you doing differently?)
- What were your expectations of the graduation process?
- How did you find bringing the service to an end for the OP you saw?)
- How have you found the completion of initial administrative tasks on the first visit (consent, fridge magnet, etc.)?

**Working within the AGE UK team**

- What are your thoughts on your work-load and the way in which you have managed it?
- Can you talk about how the support worker role fits within your service, (i.e. regularity of contact, when and how SW is involved in PCP activity and why not used in BFD)?
- How effective have you found available supervision from Age UK management?
- How have you found documenting your activities in Age UK systems
- What, if anything would you change about working in the AGE UK team? (in respect of PROSPER)

**Working with the wider practice MDT**

- How would you describe your relationship with the practice MDT team?
- How have you found building relationships with this team (*prompt* what steps have V & L taken)?
- What issues have you faced in working within an MDT with the following;
- Info sharing and communication?
- Practicalities of delivering the service?
- MDT meetings?
- Can you suggest what could be done to resolve these issues?

**Working with wider community services**

- How have you felt about your level of knowledge about local services and their availability?
- To what extent have you found that local services have been suitable and accessible to the OAs you have been working with?

**Working with Research Team**

- How have you found your interactions with the **trial team** (completion of CRFs for CTRU, support from AH, etc.)
- How have you found your interactions (obs, ints,) with **PE researchers**? (JS, AW, NK).
- Any suggestions regarding changes to the way the research aspects of the project are undertaken?

**Local/national context**

- Are you aware of any local/national issues that have impacted the service?

**Training** (already discussed in earlier interview but we’re interested in more recent views in the light of your experience of delivering the intervention)

- Do you have any further reflections on how your training could have been improved to help you deliver PROSPER?
- Which aspects would you change?
- Which aspects would you keep the same?

**Final questions**

Is there anything further that you would like to add that you think would help us with the evaluation?
