# Supplementary material for: Implementing personalised care planning for older people with frailty: a process evaluation of the PROSPER feasibility trial
Source: BMC Geriatr. 2022 Sep 16;22:760. doi: 10.1186/s12877-022-03426-4 (PMC9479257; doi:10.1186/s12877-022-03426-4)
Supplement: Supplementary file 6 — Additional file 6: Topic Guide 5. Age UK SW Exit Intervie. [file 12877_2022_3426_MOESM6_ESM.docx]

**Topic Guide 5: Age UK SW Exit Interview**

*When introducing the interview state that the questions are going to relate to the PICs overall experience of delivering the intervention throughout the delivery phase*

**Opening Questions; *general* impressions of how the PROSPER service has gone**

- What are your general impressions of how the PROSPER service as a whole has been working?
- What if anything has surprised you during the delivery phase?
- Can you talk about any barriers to implementation of service?
- What has helped with service implementation?
- What are your views on the OP that have been taking up PROSPER? (Do you feel that it has been reaching the right people and if not what could be done to target PROSPER more effectively?)
- What, if anything, can you think of that would improve the service?

**The process of delivering the Service**

**Your role**

- What were your expectations of the SW role before you started delivering PROSPER?
- How has the reality of your role matched your expectations?
- Has your role evolved during the course of delivering PROSPER, if so how?

**Organising your caseload**

- Can you tell me about the process of being allocated OP to work with? (i.e. at what point has Vicki been handing people over?)

**Working with OAs**

- Can you tell me about what elements of your training (i.e. GC, MI, Frailty, BCT) you found yourself employing? Can you talk a bit more about why things worked out this way?
- What elements of your training that you have found using less often or not at all?
- What activities you undertake with OP in your capacity as SW?
- How **involved** have you been **goal setting** and **action planning?**
- (if applicable) How were goals identified?
- (if applicable) Can you tell me about the process of action planning (i.e. to what extent was it collaborative)?
- What issues have you faced **implementing** action plans?
- Have you been involved in referring OP to services? If so, how have you found the process of referring OP?
- To what extent have your skills in delivering the PROSPER service developed over time? (If they have developed, what are you now doing differently?)
- To what extent have you been involved in the graduation process?
- (If applicable) How did you find ending your involvement with OP?
- To what extent do you think the OP generally “got” what PROSPER was all about?

**Working within the AGE UK team**

- Tell me about how you and Vicki work together (what works well and what less well).
- What do you think are the differences between your role and Vicki’s?
- What are your thoughts on your work-load and the way in which you have managed it?
- How effective have you found available supervision from Age UK management?
- How have you found documenting your activities in Age UK systems
- What, if anything would you change about working in the AGE UK team? (in respect of PROSPER)

**Working with the wider MDT**

- How would you describe your relationship with practice MDT teams?
- Can you tell me about how you have found building relationships with these teams?
- What issues have you faced in working within an MDT with the following;
- Info sharing and communication?
- Practicalities of delivering the service?
- MDT meetings?
- Can you suggest what could be done to resolve these issues?

**Working with wider community services**

- How have you felt about your level of knowledge about local services and their availability?
- To what extent have you found that local services have been suitable and accessible to the OP you have been working with?

**Working with Research Team**

- How have you found your interactions with the **trial team** (completion of CRFs for CTRU, support from AH, etc.)
- How have you found your interactions (obs, ints, Nomad) with **PE researchers**? (JS, AW, NK).
- Any suggestions regarding changes to the way the research aspects of the project are undertaken?

**Local/national context**

- Are you aware of any local/national issues that have impacted the service?

**Training** (already discussed in earlier interview but we’re interested in more recent views in the light of your experience of delivering the intervention)

- Do you have any further reflections on how your training could have been improved to help you deliver PROSPER?
- Which aspects would you change?
- Which aspects would you keep the same?

**Final questions**

Is there anything further that you would like to add that you think would help us with the evaluation?
