# Supplementary material for: Implementing personalised care planning for older people with frailty: a process evaluation of the PROSPER feasibility trial
Source: BMC Geriatr. 2022 Sep 16;22:760. doi: 10.1186/s12877-022-03426-4 (PMC9479257; doi:10.1186/s12877-022-03426-4)
Supplement: Supplementary file 8 — Additional file 8: Topic Guide 7. Older People “Limited Engager” Interview. [file 12877_2022_3426_MOESM8_ESM.doc]

**Topic Guide 7: Older People “Limited Engager” Interview**

***Intro/ “Smalltalk”-***

We’re going to focus on the PIC visit (personalised care planning). We’re interested in whether we’re taking the right approach with the intervention. Are we targeting the right people? How can it be improved? But will also ask a couple of Qs re wider PROSPER research trial.

***Initial engagement in the research project (PROSPER trial)***

- What is your understanding of why you were approached about the PROSPER trial?
- Can you tell me about why you agreed to take part in the PROSPER trial?
- How did you find the initial researcher visit (completion of baseline questionnaire/MoCA?

***Initial engagement with the Age UK Personalised Care Planning service***

- How did you first hear about the Age UK service? (PCP)
- What were your first thoughts when you first got the letter/received the call?
- Do you remember why you agreed to be visited by the PIC?
- Did you have any expectations of what the service could offer? If so what were they?

***The Guided conversation***

- Was it clear to you what the intervention was about when the PIC introduced it?
- How did you find the meeting with the PIC?
- Were there any issues discussed that were particularly important to you?
- Were there any issues that were not discussed that you felt should have been?
- How would you describe the PICs approach to the meeting?
- Why did you decide not to take up the service?
- Have you experienced a time or do you foresee a time when this service would be helpful for you? Why/why not?
- How could your experience of the service have been improved?
- What advice would you give to someone who was invited onto the programme?

***The research process (this section about researcher not PIC)***

- How did you feel about taking part in the research project to evaluate the service?
- How did you feel about a researcher sitting in on your meeting (and recording it)
- How do you feel about being asked to take part in an interview about your experience of the service?
- Do you think there are any other things that we could have done to understand your experience of the service?

***Closing questions***

- Is there anything else you feel would be useful for me to know?
- Do you have any questions for me?
