# Supplementary material for: Implementing personalised care planning for older people with frailty: a process evaluation of the PROSPER feasibility trial
Source: BMC Geriatr. 2022 Sep 16;22:760. doi: 10.1186/s12877-022-03426-4 (PMC9479257; doi:10.1186/s12877-022-03426-4)
Supplement: Supplementary file 9 — Additional file 9: Topic Guide 8. PCT Interview. [file 12877_2022_3426_MOESM9_ESM.doc]

**Topic Guide 8: PCT Interview**

*Introduction- These questions relate both to the wider research (relating to trial) and the delivery of the PROSPER service.*

**Questions about the wider research**

- Can you tell me about your involvement in the wider research and how you have found that?
- What would have made it easier for you?
- What are the learning points we need to take away from this?

**Questions about your involvement in the delivery of the PROSPER service**

- How have you found your involvement in delivering the PROSPER service?
- What are your general impressions of how the delivery of the PROSPER service has been going?
- How much did you feel you knew about the PROSPER intervention at the start (were you told enough?)
- Do you know any more now it has been going for a while?
- Can you describe your relationship with the PICs/delivery team?
- Can you talk about any barriers you’ve encountered in supporting the PICs to implement the service?
- What has helped you in supporting the PICs in implementing the PROSPER service?
- What if anything can you think of that would improve the way in which the PROSPER service is delivered?
- Is this just more work for you or do you think the PROSPER service is of value and worth supporting?
- Do you have a view on info sharing and communication with the delivery team?
