# Supplementary material for: Implementing personalised care planning for older people with frailty: a process evaluation of the PROSPER feasibility trial
Source: BMC Geriatr. 2022 Sep 16;22:760. doi: 10.1186/s12877-022-03426-4 (PMC9479257; doi:10.1186/s12877-022-03426-4)
Supplement: Supplementary file 10 — Additional file 10: Personal Independence Co-Ordinator Job Descripton. [file 12877_2022_3426_MOESM10_ESM.doc]

**PERSONAL INDEPENDENCE CO-ORDINATOR JOB DESCRIPTON**

**Job Role**

This post presents an exciting opportunity to play a pivotal ‘hands on’ role in co-ordinating health and social care services with voluntary and community sector networks. Working as part of a small Age UK team that is closely linked to primary care multi-disciplinary teams (MDTs), you will be delivering person centred support, during a discrete intervention period, to individuals aged 65+ based on the Personalised Integrated Care Programme developed by Age UK. ([https://www.ageuk.org.uk/our-impact/programmes/integrated-care/#](https://www.ageuk.org.uk/our-impact/programmes/integrated-care/))

This post is funded as part of a £2.7m research programme (PROSPER) which aims to improve the quality of life for older people with frailty by giving them choice and control over decisions about their own health and well-being.

Ideally, you will have a health or social care background (or be familiar with these areas), be resilient and work well under pressure using your creativity to co-produce solutions. You will be able to see the ‘bigger picture’, but be alert, curious and observant - so the important details of an individual’s life are not overlooked. You should be able to provide guidance to other team members supporting individuals and be prepared to work flexibly. You will also need your own transport.

This post is Band ?? full-time (37 hours) from October 2018, fixed-term for 18 months and may be suitable as a secondment opportunity.

Interviews will be held w/c Sept 3^rd^.

For informal enquiries about the post please contact??

**Main responsibilities to:**

**Individuals**

- Conducting guided conversations in the community in order to co-produce an action plan which will support the Individual to live the life they wish, to the best of their abilities, keeping their lifestyle goals at the forefront
- Liaising with relatives, carers and other agencies/ services as agreed with the individual, to ensure an integrated and personalised asset based approach to support and care
- Motivating individuals to set and achieve personal goals that will improve their quality of life and contribute to their health and wellbeing in the future
- Providing access to information & advice to individuals and their carers about a range of benefits, services and community networks that will enable them to achieve their goals
- To assist individuals in building social networks and participation in their chosen activities, hobbies and interests
- Introducing at an early stage (where appropriate) and working alongside the Support Worker to practically support the Individual in achieving their goals
- Providing a dedicated and trusted point of contact for individuals, carers and families

**Age UK**

- Undertake all Age UK mandatory training e.g. safeguarding vulnerable adults, understanding the Mental Capacity Act and Equality and Diversity
- Manage referrals and caseloads to ensure that the intervention is delivered promptly and in line with the research protocols
- Liaise closely with the Support Worker and Team Leader to manage case-loads and ensure continuity of service for individuals
- Keep accurate and up-to-date records of live cases on Charity Log.
- Develop sufficient understanding of the local health and social care landscape to co-ordinate the range of external agencies (both statutory and voluntary) which can provide assistance to individuals, carers and their families.
- Work within all relevant policies and procedures of Age UK Bradford e.g. lone working, information governance
- Report any issues that could have an impact on service delivery or quality to the Team Leader

**MDTs**

- Attend Multi-Disciplinary Team (MDT) meetings within GP practices in order to establish positive relationships and communication with referrers and colleagues across broad ranging services.
- Feedback on support being offered, raise concerns and provide non-medical contributions to an individual’s care plan.
- Monitor and update individual’s Action Plans and share with Practices
- Adhere to NHS Information Governance protocols

**Research Team**

- Undertake a comprehensive training programme to enable the delivery of the personalised care planning (PCP) service as per protocol which will include the use of guided conversations, motivational interviewing and behavioural change techniques along with developing action plans and goal setting.
- Undertake proportionate Good Clinical Practice (GCP) training in order to understand best practice in research intervention delivery
- Record all monitoring data required by the research team using pre-defined tools
- Report any deviations to the service delivery protocols to the Clinical Trials Unit via the Team Leader
- Contribute to the process evaluation and development of PCP for older people with frailty by taking part in interviews and observations as required by the research team

**Other**

- To work within all policies and procedures relevant to the PROSPER programme partnership, ensuring that individuals’ and carers’ information remains confidential.
- To undertake other duties that are consistent with the duties and responsibilities of this post as required by Age UK Bradford and the PROSPER research team

| **Person Specification** | **Essential** | **Desirable** | **Assessed by** | | |
| --- | --- | --- | --- | --- | --- |
| **Personal Independence Coordinator** |  |  | Application Form | Presentation | Interview |
| **Experience** | | | | | |
| - Experience supporting older people, particularly those living with a long term condition, frailty or caring responsibilities and/or facing loneliness and social isolation | X |  | X |  |  |
| - Experience of working alongside and in partnership with health, social care, voluntary sector organisations and groups within community settings | X |  | X |  |  |
| - Experience of using a person centred approach to identifying and meeting needs | X |  | X |  |  |
| - Experience of collaborative working as part of a delivery team | X |  | X |  |  |
| - Experience of supervision or mentorship of other team members |  | X |  |  |  |
| **Knowledge** |  |  |  |  |  |
| - Understanding of issues affecting older people, particularly those with frailty | X |  | X | X | X |
| - Understanding the prevention agenda and voluntary sector activities that support this. | X |  | X | X | X |
| - A thorough understanding of information security regulations, confidentiality and data sharing | X |  | X |  | X |
| - Awareness of the roles and responsibilities in safeguarding individuals and managing complex situations | X |  | X |  | X |
| - A working knowledge of the local health and social care landscape including access routes to the range of specialist, clinical and community resources and support available |  | X |  |  |  |
| - Knowledge of research methods and processes |  | X |  |  |  |
| **Skills** | | | | | |
| - Excellent active listening skills that demonstrate respect, understanding and sensitivity |  |  |  |  |  |
| - Ability to manage caseloads and meet targets through planning and prioritisation | X |  | X |  | X |
| - Ability to think innovatively and find creative solutions; involving other organisations and volunteers; being effective as a facilitator rather than a ‘fixer’. | X |  | X |  | X |
| - Communicate effectively and have good interpersonal skills in a range of potentially unpredictable environments and with a range of stakeholders e.g. Individuals, families, GPs and other stakeholders | X |  | X |  | X |
| - Ability to engage in reflective practise and continued learning to improve the quality and safety of the service | X |  |  |  |  |
| - Accurate record keeping, data management, recording and reporting of information. | X |  | X |  | X |
| - Ability to build on existing professional relationships and develop new ones. | X |  | X |  | X |
| - Excellent IT skills, competency in Microsoft Office applications e.g. Word, Excel | X |  | X |  | X |
| - Holder of valid GB or NI driving licence with appropriate insurance cover, including for business use. | X |  | X |  | X |
| - At least four GCSEs (Grades A to C) or equivalent must include Maths and English | X |  | X |  | X |
| - Ability to work independently but maintain a team ethos | X |  |  |  | X |
| - Use of behavioural change techniques to engage, empower and encourage individuals to make and sustain positive lifestyle changes |  | X | X |  | X |
| - Motivational interviewing skills |  | X | X |  | X |
| - Fluent in Punjab and/or Urdhu |  | X |  |  |  |
| **Other** |  |  |  |  |  |
| - Willingness to undertake comprehensive training | X |  |  |  |  |
| - Willingness to work flexibly in order to meet the requirements of the role | X |  |  |  |  |

**Interview scenario: How might a client like Mr Smith be supported to meet his goal of feeling less isolated and lonely?**

Mr Smith (80) has recently lost his wife suddenly after 50 years of marriage. He has a son and young grandchildren who live in Sheffield who he sees infrequently. He has diabetes and is suffering with depression. He also has mobility problems due to an injury whist serving in the armed forces and cannot drive. Mrs Smith, who was younger, used to do all the cooking for the couple and would frequently drive them to places of interest e.g. National Trust for days out and his son’s home. They both enjoyed quiz programmes on the television, but Mr Smith does not watch them now he does not have his wife to confer with.

1. Can you tell me a little bit about your experience of supporting older people in the community?
2. How would you define ‘person centredness’?
3. What do you think are the major challenges to partnership working?
4. What is your understanding of frailty?
5. How do you think Personalised Care Planning supports the prevention agenda?
6. What strategies would you employ to manage the caseload required in this role?
7. Accurate record keeping is essential in this post – why do you think that is?
